# Neurovascular Network Explorer 1.0: a database of 2-photon single-vessel diameter measurements with MATLAB^®^ graphical user interface

**DOI:** 10.3389/fninf.2014.00056

**Published:** 2014-05-20

**Authors:** Vishnu B. Sridhar, Peifang Tian, Anders M. Dale, Anna Devor, Payam A. Saisan

**Affiliations:** ^1^Bioengineering Undergraduate Program, University of CaliforniaSan Diego, La Jolla, CA, USA; ^2^Department of Neurosciences, University of CaliforniaSan Diego, La Jolla, CA, USA; ^3^Department of Physics, John Carroll University, University HeightsOH, USA; ^4^Department of Radiology, University of CaliforniaSan Diego, La Jolla, CA, USA; ^5^Martinos Center for Biomedical Imaging, Massachusetts General Hospital, Harvard Medical SchoolCharlestown, MA, USA

**Keywords:** database, graphical user interface, MATLAB, 2-photon microscopy, hemodynamic, cerebral cortex, arteriole, dilation

## Abstract

We present a database client software—Neurovascular Network Explorer 1.0 (NNE 1.0)—that uses MATLAB^®^ based Graphical User Interface (GUI) for interaction with a database of 2-photon single-vessel diameter measurements from our previous publication (Tian et al., 2010). These data are of particular interest for modeling the hemodynamic response. NNE 1.0 is downloaded by the user and then runs either as a MATLAB script or as a standalone program on a Windows platform. The GUI allows browsing the database according to parameters specified by the user, simple manipulation and visualization of the retrieved records (such as averaging and peak-normalization), and export of the results. Further, we provide NNE 1.0 source code. With this source code, the user can database their own experimental results, given the appropriate data structure and naming conventions, and thus share their data in a user-friendly format with other investigators. NNE 1.0 provides an example of seamless and low-cost solution for sharing of experimental data by a regular size neuroscience laboratory and may serve as a general template, facilitating dissemination of biological results and accelerating data-driven modeling approaches.

## Introduction

Recent advances of 2-photon microscopy have enabled cellular and sub-cellular level measurements of live tissue with unprecedented spatial and temporal resolution, sensitivity, and specificity. However, due to its microscopic nature, many individual measurements at different points in space and time are usually required to reconstruct the phenomenon under investigation. For example, one might measure calcium activity at many points along the dendritic tree to examine the role of calcium excitability in summation of synaptic inputs (Larkum et al., 2009). Likewise, measurements of activity across many individual neuronal cell bodies are required for estimation of the ensemble spiking response in a cortical circuit (Gobel et al., 2007). Further, depending on the specific 2-photon contrast, a single measurement on its own might be too “noisy,” requiring group-averaging for drawing biological conclusions (Devor et al., 2011).

With the vast number of individual microscopic measurements, there is a common need for structuring of the data in a way that allows automation of computational tasks and extraction of statistical properties for hypotheses testing. Furthermore, organized as a database, an experimental study can be shared with the research community allowing a close inspection of the data and facilitating the use of experimental data for modeling.

Here we describe such a database that contains stimulus-induced single-vessel diameter change data from a published 2-photon microscopy study in the rat primary somatosensory cortex (SI) *in vivo* from our laboratory (Tian et al., 2010). We have created a database client software—Neurovascular Network Explorer 1.0 (NNE 1.0)—that uses MATLAB^®^ graphical user interface (GUI) for browsing the database, simple manipulation of the retrieved records (averaging, peak-normalization), and export of results in a numerical format. NNE 1.0 runs under any modern Windows operating system, and does not require MATLAB license or knowledge of MATLAB computing language. For users with experience in MATLAB, we provide the script used to create NNE 1.0. Given the appropriate data structure and naming conventions, the user can utilize this script to database and interactively display their own time series data (e.g., time-courses of calcium imaging signals across individual neurons).

NNE 1.0 and the associated database provide an example of how experimental data from a regular size neuroscience laboratory can be made freely available with a minimal need for extra resources. A key requirement is exercising the use of fixed format arrays to capture individual data records.

## Methods

**Figure 1:**
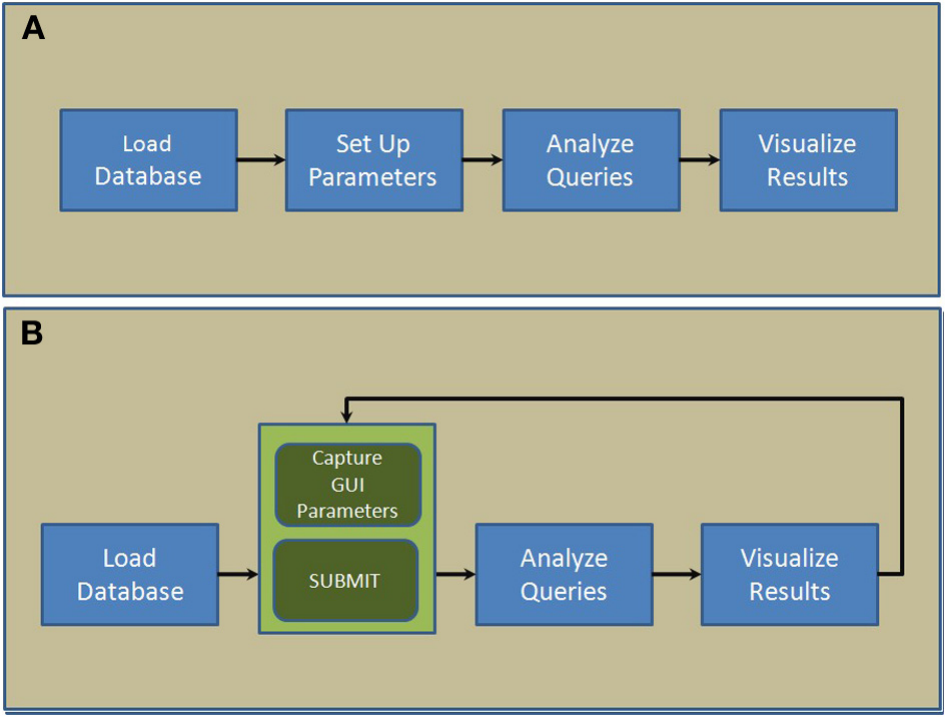
**Scripting architecture of the 2-step paradigm used to develop NNE 1.0. (A)** The first step where 4 core modules are put together sequentially. **(B)** The second step where a new GUI-based module captures parameters and displays results interactively.

NNE 1.0 database client software was developed in MATLAB in 2 steps. In the first step, 4 core modules: “Load database,” “Set up parameters,” “Analyze queries,” and “Visualize results,”—were designed and put together sequentially as shown in Figure 1A. Next, we replaced the second module, “Set up parameters” with a GUI (Figure 1B). This interface captures user parameters in 3 panels (see Results), displays, and exports results. This 2-step paradigm allows advanced MATLAB users to quickly develop complex data query and analysis modules and combine them with the GUI to capture parameters and display results if they were to customize the source code for their own data.

With NNE 1.0, the user specifies search criteria through the GUI, and the database client displays individual as well as group-averaged time-course and scatter plots for the selected data subset through modules “Analyze queries” and “Visualize results.” In addition, the user can click on individual time-courses to extract specific parameters associated with the selected database entry. Finally the user can export search results in MATLAB and Excel formats.

To make NNE 1.0 available to users without a MATLAB license, we also generated an x86 binary executable program using MATLAB compiler. The executable file includes all necessary run-time components and runs as a standalone program. To use it, one needs to download and install the MATLAB Compiler Runtime (MCR) included in our download package. Hence, the database client can be used either as a MATLAB script or as a standalone program. The requirement is sufficient amount of memory (minimum of 2GB of system RAM), modern Windows OS, and internet connection to download and access the client software and relevant database files. Full database format and field access details are outlined in the source code (corresponding to the architecture in Figure 1B), which can be downloaded from our academic website *nil.ucsd.edu* under “*Use our data*.”

## Results

### Database structure and naming conventions

**Figure 2:**
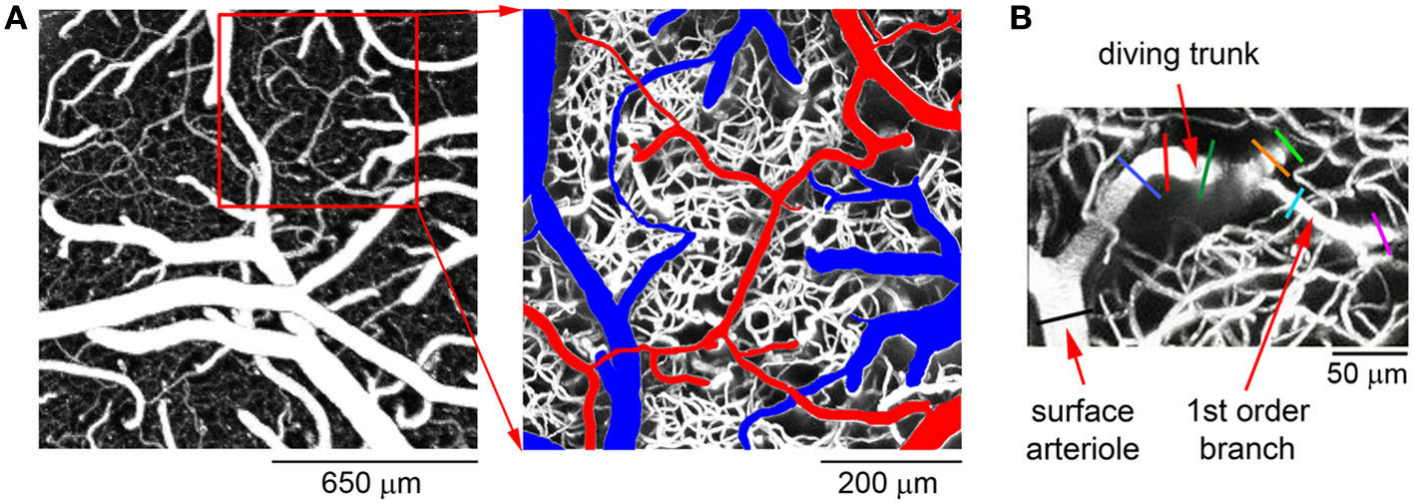
**A typical example of the cortical microvasculature. (A)** Left: an image of the cortical microvasculature calculated as a maximum intensity projection (MIP) of an image stack 0–300 μm in depth using a 4× objective. Individual images were acquired every 10 μm. Right: a detailed view of the region within the der square on the left. Surface arteries and veins are indicated in red and blue, respectively. **(B)** MIP of an image stack 0–300 μm in depth featuring a single diving arteriolar tree. For this specific example, diameter changes were measured at the parent surface arteriole close to the diving point (black line), the diving trunk (blue, red, and dark green lines) and along 2 lateral branches at different cortical depths (orange, green, cyan, and purple lines).

In our original data paper, we reported diameter changes of diving arterioles and their branches from 0 to 550 μm below the cortical surface in the rat SI in response to forepaw sensory stimulation (Tian et al., 2010). Figure 2A shows a typical view of the cortical microvasculature, after exposing the cortical tissue, obtained with 2-photon microscopy. Arteriolar “tree” is used to refer to the diving segment (trunk) and lateral branches that can be classified by branching order. Each arteriolar tree has a “parent” surface arteriole—a pial arteriole that is the origin of the diving trunk. For the specific example shown in Figure 2B, stimulus-induced diameter changes were measured at multiple locations, including the parent surface arteriole close to the diving point (black line in Figure 2B), the diving trunk (blue, red, and dark green lines), and its lateral branches at different cortical depths (orange, green, cyan, and purple lines). In total, the database includes diameter change time-courses of 87 arteriolar trees measured at 398 different locations.

Ten stimulus trials were averaged for each location (Tian et al., 2010), and the database entry corresponds to a trial-averaged diameter timecourse at a specific measurement location along one of the measured trees. In addition to the diameter timecourse, each database entry holds an array of descriptive parameters, such cortical depth and branching order at the measured location (Table 1). The data are organized as a MATLAB “structure,” which can be understood as a matrix, namely, vdb (stands for “vascular database,”) consisting of 399 rows and 11 columns. The first row vdb{1,:} contains the names of parameters in each column as defined in Table 1. Each of the remaining 398 rows corresponds to a particular measurement location. For example, vdb{i,7} is the onset time of dilation at the i*th* measurement location. The database structure, vdb, is the input to NNE 1.0, which is designed to take any MATLAB database structure of the same format and run analysis and data query to produce the computational results discussed in the next section.

**Table 1:** **Database structure and naming conventions**.

	**Parameter name**	**Description**
vdb{i,1}	Subject ID (Date)	Unique subject identifier
vdb{i,2}	Tree ID	Unique arteriolar tree identifier
vdb{i,3}	Time	A time vector for the “Timecourse,” see below. “Time” is in seconds starting from the stimulus onset
vdb{i,4}	Timecourse	Arteriolar diameter change as a function of time. “Timecourse” is expressed as percent change from the baseline. “Time” and “Timecourse” have the same size
vdb{i,5}	Normalized timecourse	“Timecourse” normalized to the peak amplitude
vdb{i,6}	Branching order	Branching order at the measurement location. The possibilities are: surface arteriole, diving trunk, 1st order branches, and higher order branches
vdb{i,7}	Onset time	The estimated onset time of dilation. For details, see the inset of Figure 1D in Tian et al. (2010)
vdb{i,8}	Time-to-peak	The time when vessel diameter reaches its peak dilation
vdb{i,9}	Cortical depth	The depth of the measurement below the cortical surface
vdb{i,10}	Baseline diameter	Vessel diameter prior to stimulus onset
vdb{i,11}	Peak amplitude	Peak dilation expressed as percent change from the baseline

### Graphical user interface of NNE 1.0

**Figure 3:**
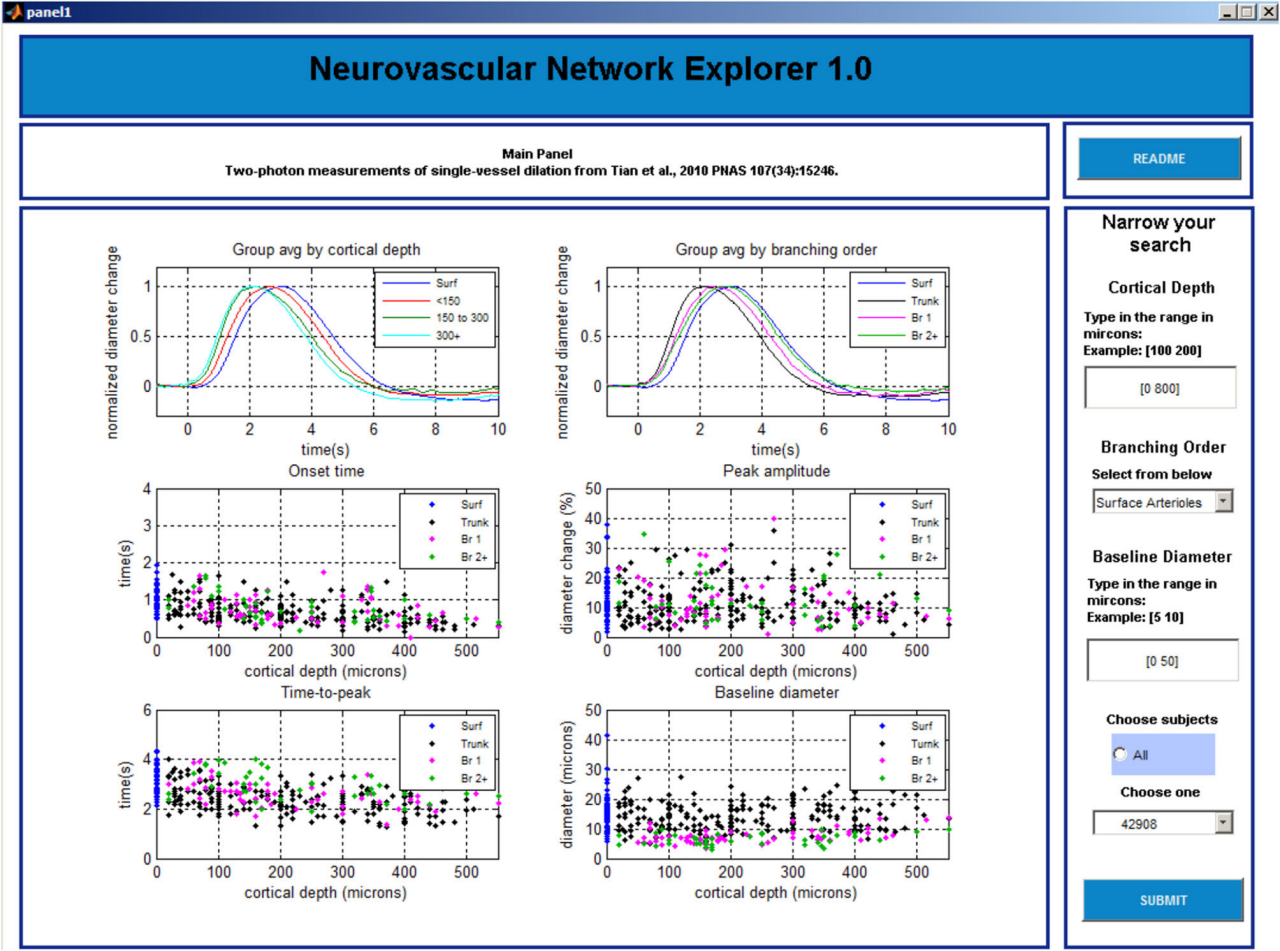
**GUI, panel 1**.

**Figure 4:**
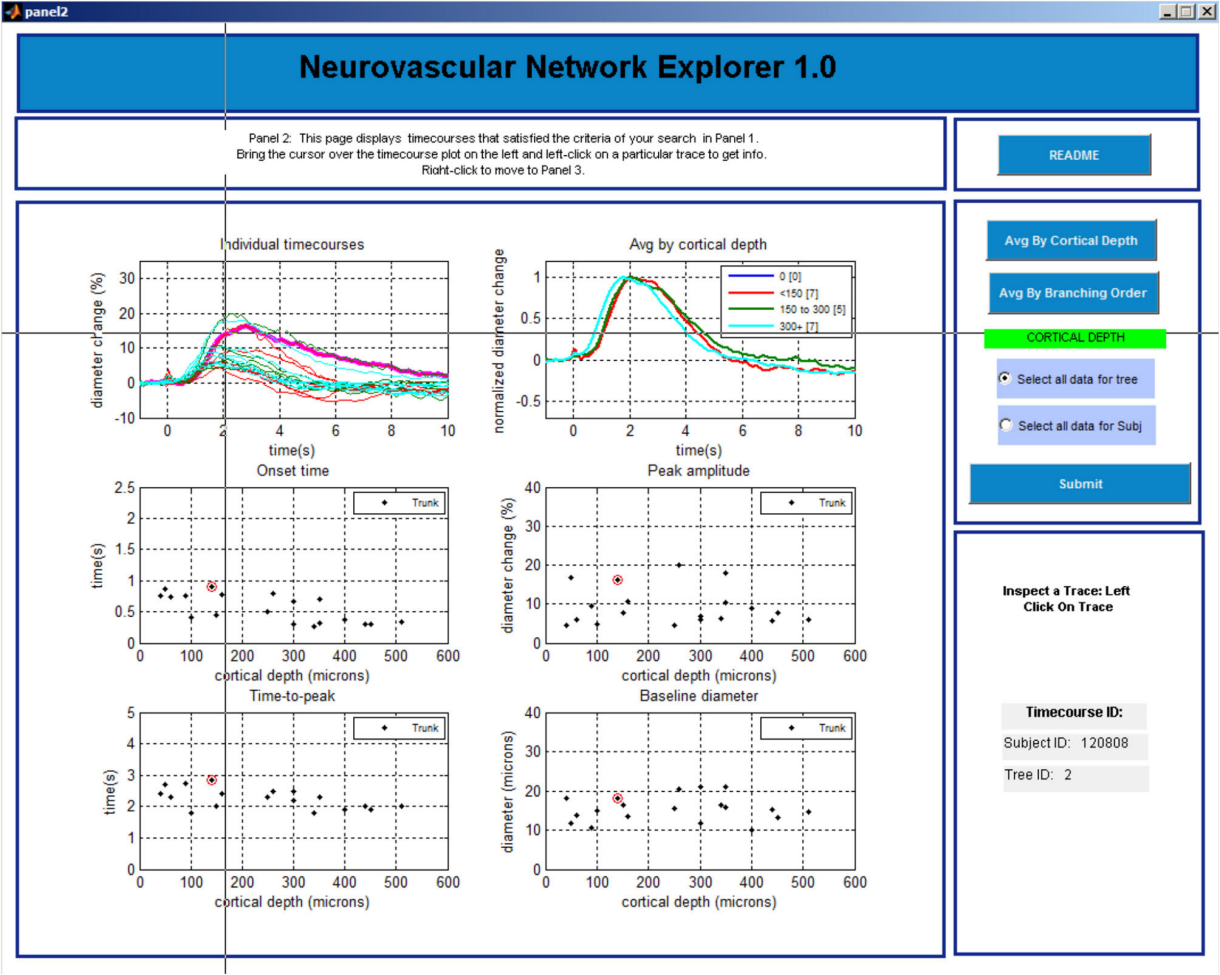
**GUI, panel 2**.

**Figure 5:**
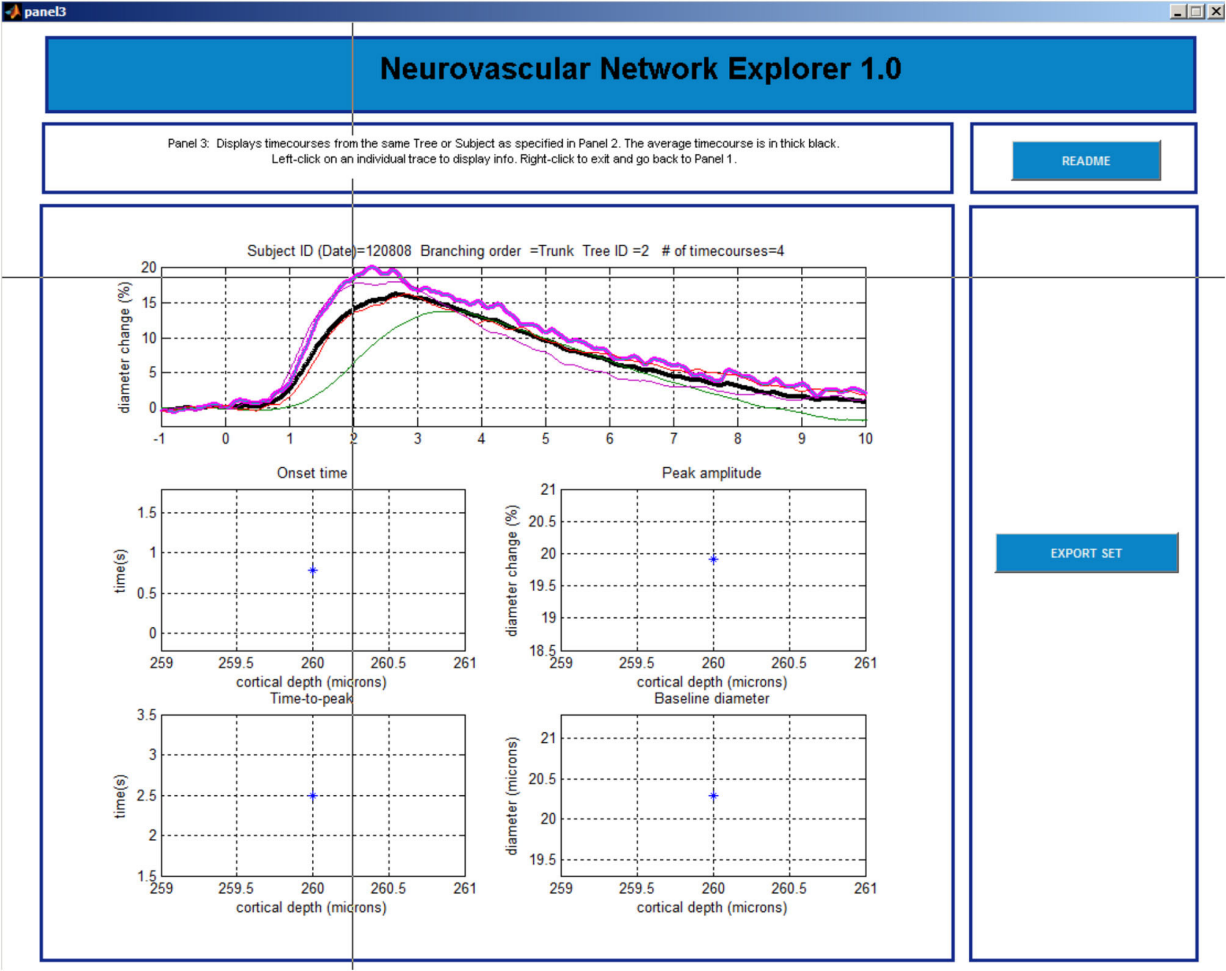
**GUI, panel 3**.

The GUI consists of 3 data query and visualization panels (Panels 1–3, Figures 3–5), where the progression from general to specific data pruning occurs. The user can select, manipulate, visualize, and export specific data of interest based on parameters such as cortical depth, branching order, and baseline diameter as well as Subject and Tree IDs. All 3 panels display: (1) dilation timecourses (stimulus-induced diameter change as a function of time), and (2) scatter plots of onset time, time-to-peak, peak amplitude, and baseline diameter as a function of cortical depth. Next, we discuss each panel in details.

#### Panel 1

The main window of Panel 1 is not interactive and gives an overview of the data (Figure 3). The top graphs display group-averaged, peak-normalized dilation time-courses for the entire dataset. Averaged time-courses are grouped by cortical depth (top left: surface; 0–150 microns; 150–300 microns; and deeper than 300 microns) and branching order (top right: surface; diving trunks; 1st order branches, and higher order branches). The remaining four graphs display scatter plots of onset time, time-to-peak, peak amplitude, and baseline diameter as a function of cortical depth for the entire dataset.

The left column of Panel 1 (Figure 3) allows the user to enter the following search parameters:

- Cortical depth range, entered in plain text
- Branching order, selected from one of the 4 options: surface arterioles, diving trunk, first order branches, and higher order branches (2–4)
- Baseline diameter range, entered in plain text
- Subject selection: all subjects or an individual subject from the drop-down list

Once all selections are made, a click on the “SUBMIT” button brings up Panel 2.

At any time, the user can evoke a help document by clicking on the “README” button located in the upper right corner on this and following panels.

#### Panel 2

The main window is initially blank. The user needs to choose in the column on the right whether to perform group average by cortical depth or branching order and click “SUBMIT” button before 6 plots are displayed in the main window (Figure 4). The top left plot shows a family of overlaid individual time-courses that satisfy the search criteria specified on Panel 1. The top right plot shows peak-normalized averages. Four scatter plots below display the onset time, time-to-peak, peak amplitude, and baseline diameter as a function of the cortical depth.

The user can select a particular entry by left clicking on a time-course in the top left plot. Subsequently, the time-course will be highlighted, along with the corresponding onset time, time-to-peak, peak amplitude, and baseline diameter in the other plots. In addition, the corresponding Subject and Tree IDs will be displayed in the lower right column.

Next, the user can explore all entries associated with this Subject ID (all measurements within this individual subject) or Tree ID (all measurements along this specific arteriolar tree). The default option is “Select all data for tree.” A right click anywhere brings up Panel 3 that shows all individual time-courses for that Tree ID. If inspection of all measurements within a subject is desired, the user needs to return to Panel 2 (by closing Panel 3 window [x]) and chose “Select data for subject” followed by a click on the “SUBMIT” button. The user can also go back to Panel 1 by closing this window [x].

#### Panel 3

This last panel consists of 5 plots (Figure 5). The top plot shows all individual time-courses that satisfy the search criteria specified on Panel 2. The user can select a particular entry by left clicking on the time-course. The 4 graphs below will then display the onset time, time-to-peak, peak amplitude, and baseline diameter associated with this database entry. The user can export a single time-course, a family of time-courses that belong to the chosen subject, or a family of time-courses that belong to the chosen tree in MATLAB format (.mat) or Excel format [comma separated values (.csv) files]. The user can return to Panel 1 by a right click and repeat the search.

### Comparison with mainstream databasing tools

Compared with Structured Query Language (SQL) and SQL-like relational database management systems that focus primarily on large-scale database design and management elements, our MATLAB based GUI approach has 2 advantages. First, it is simpler to implement and offers more intuitive scripting structure and a flexible data format. Therefore, it has less learning and developmental overheads and is more user-friendly. Second, the databasing GUI elements can be seamlessly linked to other computational routines such as data analysis scripts written in MATLAB as shown in Figure 1. providing more flexibility. Here, we have created a variety of computational routines such as data resampling, temporal signal alignment, and data visualization by adapting code directly from earlier homebuilt MATLAB routines. Thus, MATLAB provides a more efficient database platform in the environment of constantly evolving data processing stream, which is inherent in novel experimental projects that employ cutting edge experimental technologies.

## Conclusions and further directions

In our laboratory, we routinely receive requests to provide experimental data in a numerical form. These requests come mainly from investigators looking for empirical measurements to constrain computational models. These ongoing requests have served as a key motivation to create NNE 1.0. For the internal use in our laboratory, NNE 1.0 has provided an easy and interactive access to the data at any time by anyone, with or without MATLAB knowledge.

The current database structure can be easily expanded to hold pointers to additional supplementary information (e.g., image stacks showing the position of the imaged arteriole relative to the larger vascular network or simultaneously performed electrophysiological recordings), additional search engines, and computational algorithms for extraction of other properties (e.g., the absolute dilation amplitude in microns). Since the original publication of these data (Tian et al., 2010), we have conducted a follow-up study in the mouse with deeper penetration and larger number of samples (Tian et al., 2012). These data will be included in the next release (NNE 2.0). Last but not least, the present database may serve as a seed for sharing of large data sets across different research groups to generate a central repository that would parallel the existing “neuronal” databases such as the NeuroMorpho.org (Ascoli et al., 2007), in particular if the data were collected and processed in a standardized way. Standardization is an important requirement when data are pooled across studies for modeling of experimental results.

With the increasing sophistication of microscopic measurement tools, there is a growing recognition of complex interactions between the cellular elements of cerebral tissue, including not only neurons but also astrocytes and vascular networks. Integration of these data across multiple measurement modalities and spatial and temporal scales requires a comprehensive computational framework, implying cross-disciplinary collaboration between experimentalists and theoreticians. We believe that NNE 1.0 will find utility as a template for seamless and low-cost solution for sharing of experimental data, facilitating dissemination of biological results and accelerating data-driven modeling approaches.

### Conflict of interest statement

The authors declare that the research was conducted in the absence of any commercial or financial relationships that could be construed as a potential conflict of interest.
